# Diagnosis of vascular invasion in pancreatic ductal adenocarcinoma using endoscopic ultrasound elastography

**DOI:** 10.1186/s12876-020-01228-9

**Published:** 2020-03-30

**Authors:** Kenta Yamada, Hiroki Kawashima, Eizaburo Ohno, Takuya Ishikawa, Hiroyuki Tanaka, Masanao Nakamura, Ryoji Miyahara, Masatoshi Ishigami, Yoshiki Hirooka, Mitsuhiro Fujishiro

**Affiliations:** 1 Department of Gastroenterology and Hepatology, Nagoya University Graduate School of Medicine, 65 Tsuruma-cho, Showa-ku, Nagoya, 466-8550 Japan; 2 Department of Liver, Biliary Tract and Pancreas Diseases, Fujita Health University, 1-98 kutsukake-cho dengakekakubo, Toyoake, 470-1192 Japan

**Keywords:** Endoscopic ultrasound, Endoscopic ultrasound elastography, Pancreatic ductal adenocarcinoma, Diagnosis

## Abstract

**Background:**

Vascular invasion is an important criterion for resectability and deciding the therapeutic strategy for pancreatic ductal adenocarcinoma (PDAC), but imaging diagnosis is currently difficult. Endoscopic ultrasound (EUS) elastography (EG) images have band-like artifacts on the border between tumor and vessel due to different movement if the tumor is not connected to the vessel, i.e., no invasion. Based on this phenomenon, we assessed the usefulness of EUS-EG in the diagnosis of vascular invasion in PDAC.

**Methods:**

The subjects were 44 out of 313 patients with PDAC who underwent EUS between January 2015 and November 2018, followed by surgery, no chemotherapy or radiotherapy, and pathological evaluation. Diagnostic accuracies of vascular invasion using dynamic computed tomography (CT), EUS B-mode and EUS-EG were compared with histopathological diagnosis.

**Results:**

In 44 subjects (48 sites) who underwent both dynamic CT and EUS-B mode, the sensitivity, specificity and accuracy were 0.733, 0.697 and 0.708 on dynamic CT (48 sites); 0.733, 0.606 and 0.646 in EUS B-mode (48 sites); and 0.917, 0.900 and 0.906 in EUS-EG (32 sites). In 27 subjects (29 sites) with a tumor contacting a vessel with no vascular obstruction or stenosis on dynamic CT, the sensitivity, specificity and accuracy were 0.556, 0.750 and 0.690 on dynamic CT; 0.667, 0.700 and 0.690 in EUS B-mode; and 0.889, 0.850 and 0.862 in EUS-EG.

**Conclusions:**

These results suggest that EUS combined with EG improves diagnostic performance of vascular invasion in PDAC, especially in cases of which vascular invasion cannot be clearly assessed by dynamic CT.

## Background

Criteria for resectability of pancreatic ductal adenocarcinoma (PDAC) are used in determination of the therapeutic strategy. The National Comprehensive Cancer Network (NCCN) Guidelines divide invasion into that in the arterial and portal systems [1]. The Guidelines recommend resection for a tumor with vascular invasion based on computed tomography (CT), but it is sometimes difficult to judge the presence of invasion. Endoscopic ultrasonography (EUS) has good spatial resolution, and is used for qualitative diagnosis of pancreatobiliary disease and differential diagnosis of benign and malignant disorders. However, the NCCN Guidelines state that EUS complements CT for diagnosis of disease stage in selected patients, but provide no description of findings and evaluation procedures.

Tissue elasticity can be imaged noninvasively by elastography, as shown in the mammary, thyroid and prostate gland [2–6]. Transabdominal ultrasonography elastography is used to diagnose hepatic fibrosis [7]. We have reported that transabdominal ultrasound elastography is useful for differential diagnosis of pancreatic disease and presumed reflux esophagitis, and that EUS elastography (EUS-EG) can predict pancreatic fibrosis and the risk for postoperative pancreatic fistula [8–11]. Giovannini et al. showed that elastography can be used for differential diagnosis of pancreatic tumor by classifying tumors into 5 grades based on skewness and signal distribution [12].

An assessment of vascular invasion of PDAC using elastography has not been performed [13]. Therefore, the primary endpoint in this study was to compare diagnostic performance for vascular invasion among dynamic CT, EUS B-mode, and EUS-EG. The secondary endpoint was to examine EUS-EG for diagnosis of vascular invasion in cases that were difficult to diagnose using dynamic CT.

## Methods

### Patients

The subjects were patients with PDAC at our hospital who underwent EUS between January 2015 and November 2018, followed by surgery without chemotherapy or radiotherapy, and a detailed pathological evaluation. We excluded the subjects in which the tumor was clearly separated from the vessels by dynamic CT. Dynamic CT was performed using a pancreatic protocol in all subjects. The EUS procedure was performed by one of three experienced endosonographers (> 250 EUS cases per year).

### Devices and procedures

The ultrasound observation system and endoscopes used in the study were an Arietta 850 (Hitachi-Aloka Medical, Ltd., Tokyo, Japan) and a GF-UE260 or GF-UCT260 (Olympus Co., Ltd., Tokyo, Japan); an EU-ME2 Premier Plus and GF-UE260 (all Olympus Co., Ltd., Tokyo, Japan); a Hi Vision Ascendus (Hitachi-Aloka Medical, Ltd., Tokyo, Japan) and EG-3670URK (Pentax Co., Ltd., Tokyo, Japan); and a Sonart SU-1 and EG-580UR or EG-580UT (all Fujifilm Co., Ltd., Tokyo, Japan).

When performing EUS-EG, the EUS probe was applied to the gut wall just exerting the pressure needed for an optimal and stable B-mode image at 7.5 MHz. The region of interest (ROI) for the elastographic evaluation was manually selected so that the lesion and the concerned vessels are centered in the ROI area. Because elastography images tend to show rapidly changing colors, an image that was stable for at least 5 s was required for evaluation of vascular invasion. The elastography images were captured continuously for at least 5 s in each case, and the still images obtained during this period were manually stored. Among them, one endoscopist (HK) who did not know the clinical characteristics or pathological results selected a single elastography image which showed the shortest distance between the tumor and the blood vessel on the referential ultrasound image. The diagnosis of vascular invasion by EUS-EG was made based on this single image for each site.

### Definition

**Fig. 1:**
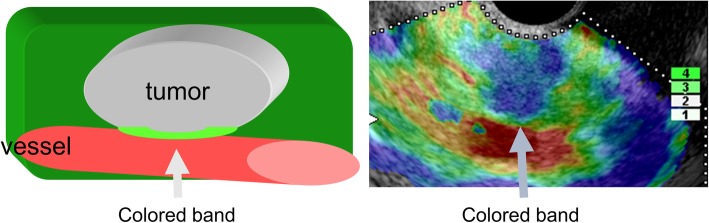
Colored band. The tumor is imaged in blue and the vessel in red. A green band (colored band; arrow) is observed between the tumor and vessel in vascular invasion-negative cases

**Fig. 2:**
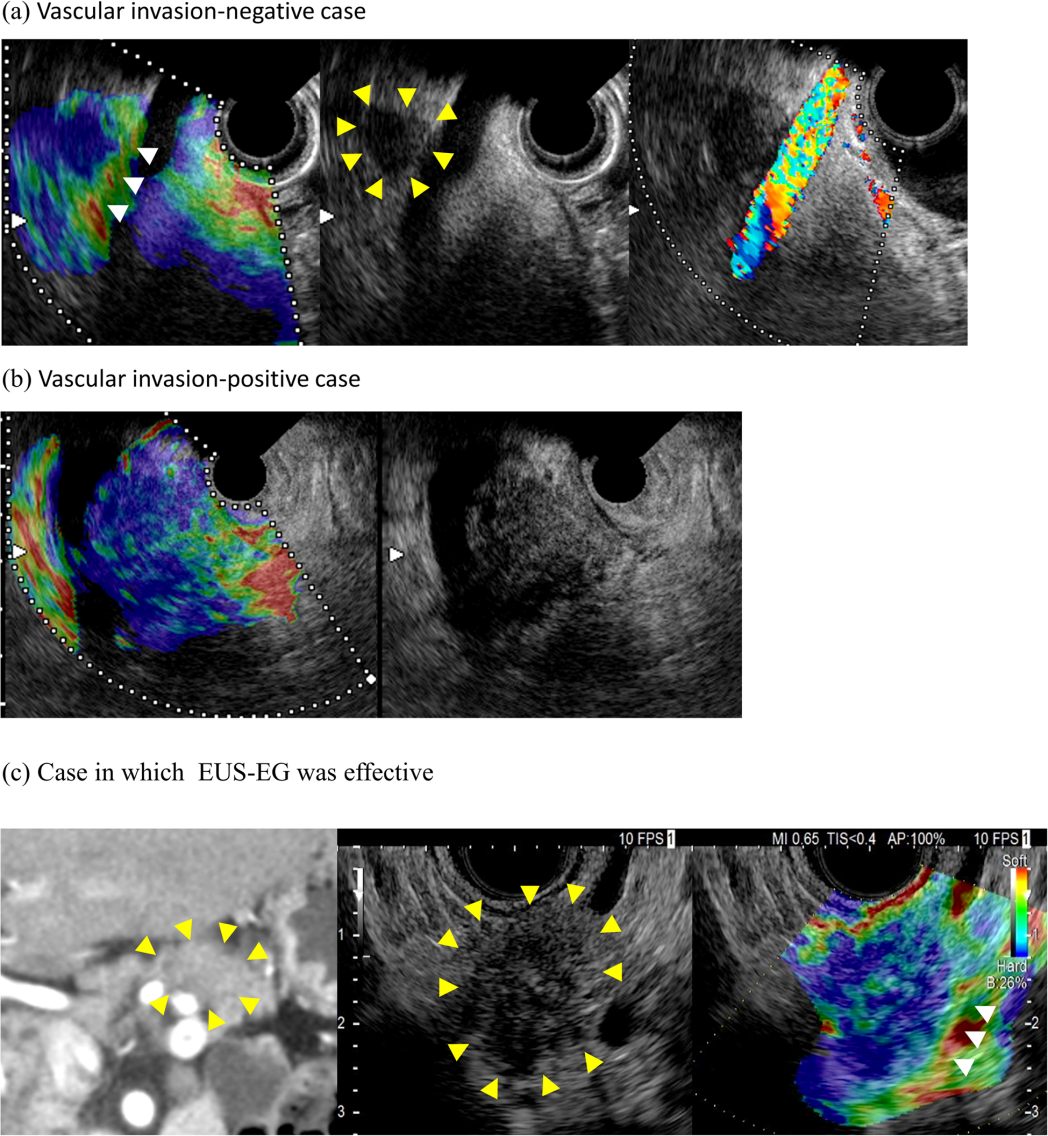
Diagnosis of vascular invasion by Endoscopic ultrasonography Elastography (EUS-EG). **a** Vascular invasion-negative case. Starting from the left: EUS-EG, EUS B-mode and color Doppler. In B-mode, vascular invasion of the tumor (yellow arrow) is unclear in EUS B-mode image, whereas the colored band (arrow) in EUS-EG image clearly identifies a vascular invasion-negative. **b** Vascular invasion-positive case. Starting from the left: EUS-EG and EUS-B-mode. In EUS-B-mode, vascular invasion of the tumor is not clear, whereas the absence of a colored band in EUS-EG image permits diagnosis of the site as vascular invasion-positive. **c** Vascular invasion-negative case in which EUS-EG was effective. Starting from the left: CECT, EUS B-mode and EUS-EG. In CECT, the tumor (yellow arrow) appears to invade the splenic artery. In B-mode, vascular invasion of the tumor (yellow arrow) is unclear in EUS B-mode image, whereas the colored band (arrow) in EUS-EG image clearly identifies a vascular invasion-negative. Pathologically, this case did not infiltrate the splenic artery. CECT: contrast enhanced computed tomography, EUS: endoscopic ultrasonography, EUS-EG: endoscopic ultrasonography elastography

The definition of vascular invasion diagnosis in EUS-EG was as below. Elastography images of the hardness or softness of biological tissues are determined by displacement of points in ultrasound upon compression of tissues. If two tissues with different hardness contact each other but are not fixed, the border significantly moves after pushing the two tissues at the same compression level. Elastography recognizes tissues showing relative movement as softer than surrounding tissues, and red, yellow and green bands are imaged as artifacts. In contrast, if two tissues are fixed, the border moves simultaneously, resulting in no artifact band at the border. In this study, we refer to artifact bands at the border as colored band. Based on this principle, lesions with and without colored band between the tumor and vessel were defined as not having and having vascular invasion, respectively (Figs. 1 and 2).

The definition of vascular invasion in EUS B-mode was based on one of three conditions being present, as in previous studies: (I) a missing portal vein and developing collateral circulation surrounding the pancreas; (II) a tumor in the intravascular space; and (III) an abnormal vessel missing a hyperechoic layer vascular surface. A subject with none of these three findings was defined as vascular invasion-negative [14].

Both EUS-EG and EUS B-mode findings were retrospectively reviewed by two gastroenterologists (YK and IT) who were experts of EUS with experience of more than 1000 procedures without the information of CT or final results in a blinded fashion.

The interobserver variability of EUS B-mode and EUS-EG was assessed by calculating the κ-coefficient after two blinded readers had made their individual independent reading. The two readers reassessed the image of that yielded discrepant finding together to reach an agreement.

Findings on dynamic CT were assessed by experienced two radiologists based on the NCCN Guidelines. Vessel margin irregularity or tumor intrusion into the periarterial fat plane with the tumor lying in juxtaposition to the vessel was assessed as vascular invasion in the arterial system [15–17]. The presence of venous occlusion, flattening or narrowing; apposition with concavity toward the vessel lumen; or a circumferential apposition > 180° was assessed as vascular invasion in the portal system [15–20].

Diagnostic performance by modality was retrospectively compared with histopathological results. Positive pathological vascular invasion was defined as tumor invasion into the vascular wall, including the adventitia [21]. Those with a tumor on the detached surface of the portal vein were regarded as positive for portal vein invasion. The diagnostic ability of dynamic CT, EUS B-mode, and EUS-EG were compared and the performance of each probe (radial or linear) used in EUS was also compared.

Difficult diagnosis site group was defined as those in which tumors and blood vessels were in contact and not had vascular obstruction or stenosis on dynamic CT. In this group, dynamic CT, EUS B-mode, and EUS-EG were performed in all subjects. Diagnostic ability of vascular invasion in the group of difficult diagnosis sites was retrospectively compared between dynamic CT and EUS B-mode and EUS-EG. The diagnosis of vascular invasion by dynamic CT in this group was made based on other findings than obstruction or stenosis. We also compared the diagnostic performance of PV, SMV, and SMA, which are important for diagnosis of T staging and resectability, but difficult to evaluate in EUS B-mode.

This study was approved by the ethical committee of our hospital (approval number 2014–0399) and performed according to the guidelines in the Helsinki Declaration for biomedical research involving human subjects (Clinical trial registration number: UMIN 000016497).

### Statistical analysis

Statistical analyses were performed using SPSS Statistics 25.0 (SPSS, Inc., Chicago, IL, USA). To evaluate the diagnostic performances for vascular invasion by each modality, the sensitivity, specificity, positive predictive value (PPV), negative predictive value (NPV), and accuracy were calculated with 95% confidence interval (95%CI). Interobserver agreements of both EUS-EG and EUS B-mode findings were assessed using κ statistics. Depending on κ values, agreement was considered as slight (.01–.20), fair (.21–.40), moderate (.41–.60), substantial (.61–.80), or almost perfect (.81–1.00).

## Results

**Fig. 3:**
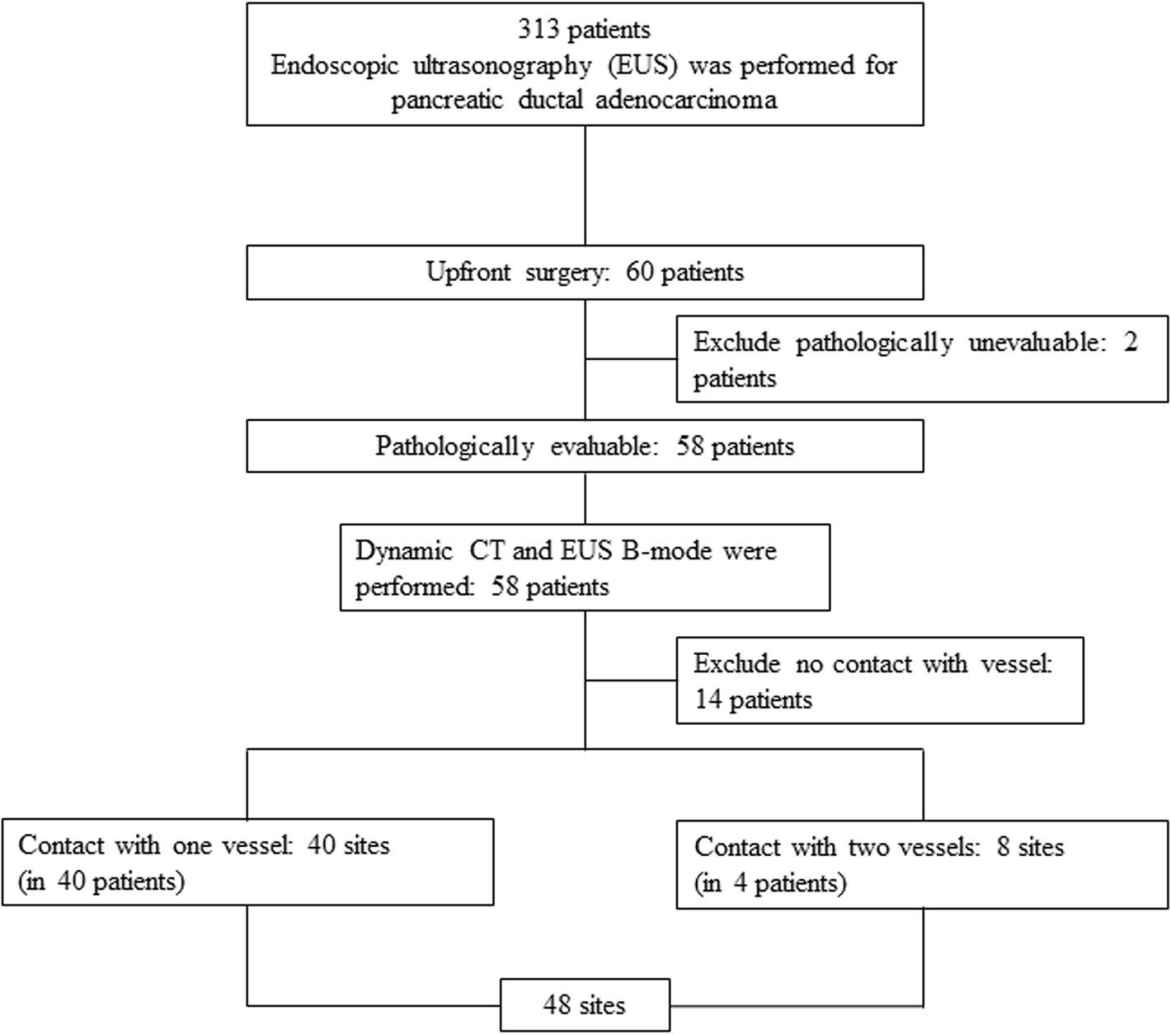
Flowchart of patients’ selection. EUS: endoscopic ultrasonography, EG: elastography, CT: computed tomography

Of 313 patients with PDAC who underwent EUS between January 2015 and November 2018 in our hospital, 60 patients underwent surgery without chemotherapy or radiotherapy and 58 in whom vascular invasion was pathologically evaluated. Of these, 14 patients with distant tumors from blood vessels were excluded and 44 patients were subjects of this study. The median age was 71 (range 44–84) years old and the male to female ratio was 30:14. The lesion sites were the pancreatic head in 19, pancreatic body in 12 and pancreatic tail in 13. The median tumor diameter was 20 (range 9–40) mm. Subtotal stomach-preserving pancreatoduodenectomy was performed in 17 subjects (of which 8 subjects were resected with portal vein, 1 subject was resected with right hepatic artery), 23 subject s were distal pancreatectomy, and 4 subject s were total pancreatectomy (Table 1). The ultrasound observation system used Arietta 850 in 10 subjects (a linear instrument /a radial instrument: 1/9), EU-ME2 Premier Plus in 2 subjects (all radial instrument), Hi Vision Ascendus in 8 subjects (all radial instrument), Sonart SU-1 in 24 subjects (a linear instrument /a radial instrument: 14/10). Of these 44 subjects, 40 and 4 had tumors that contacted one and two vessels, respectively, on dynamic CT findings. A total of 48 sites were assessed (Fig. 3).

**Table 1:** Patients’ characteristic (*n* = 44) and assessed vessels

Median age (range)	71 (44–84)
Gender (Male:Female)	30:14
Location (Ph:Pb:Pt)	19:12:13
Median tumor size (mm)(range)	20 (9–40)
Surgical procedure	
SSPPD	17
DP	23
TP	4
Assessed vessels (Pathological vascular infiltration positive)	
SMA	1 (0)
SPA	10 (2)
GDA	1 (1)
RHA	1 (1)
PV	11 (3)
SMV	7 (1)
SPV	17 (7)

*Ph* pancreas head, *Pb* pancreas body, *Pt* pancreas tail, *SSPPD* subtotal stomach-preserving pancreatoduodenectomy, *DP* distal pancreatectomy, *TP* total pancreatectomy, *SMA* superior mesenteric artery, *SPA* splenic artery, *GDA* gastroduodenal artery, *RHA* right hepatic artery, *PV* portal vein, *SMV* superior mesenteric vein, *SPV* splenic vein

The assessed blood vessels were the portal vein (PV) in 11 sites, superior mesenteric vein (SMV) in 7, splenic vein (SPV) in 17, superior mesenteric artery (SMA) in 1, splenic artery (SPA) in 10, gastroduodenal artery (GDA) in 1, and right hepatic artery (RHA) in 1. Among them, pathological vascular infiltration was positive in 5 sites of PV, 10 sites of SMV, 7 sites of SPV, 0 sites of SMA, 2 sites of SPA, 1 site of GDA, 1 site of RHA (Table 1). In difficult diagnosis sites group, the assessed blood vessels were PV in 9 sites, SMV in 3, SPV in 9, SMA in 1, SPA in 5, GDA in 1, and RHA in 1, respectively. Dynamic CT using a pancreatic protocol was performed in all 44 subjects. Vascular invasion was assessed by EUS-B mode and EUS-EG in 44 and 27 subjects, respectively. Seventeen subjects did not undergo EUS-EG.

The interobserver agreements of EUS B-mode and EUS-EG findings for vascular invasion were moderate (κ = 0.542) and substantial (κ = 0.625). The results of discrepancies among observers were 11 sites out of 48 sites in B-mode and 6 sites out of 32 sites in EUS-EG.

The sensitivity, specificity, PPV, NPV, and accuracy (95% CI) for vascular invasion in the 44 subjects (48 sites) were 0.733 (0.525–0.880), 0.697 (0.602–0.763), 0.524 (0.375–0.628), 0.852 (0.736–0.933), and 0.708 (0.578–0.800) on dynamic CT; 0.733 (0.524–0.882), 0.606 (0.511–0.673), 0.458 (0.327–0.511), 0.833 (0.702–0.926), and 0.646 (0.515–0.739) in EUS B-mode; and 0.917 (0.723–0.983), 0.900 (0.784–0.940), 0.846 (0.667–0.908), 0.947 (0.825–0.990), and 0.906 (0.761–0.956) in EUS-EG (32 sites) (Table 2). In the radial instrument, the sensitivity, specificity, PPV, NPV, and accuracy for vascular invasion were 0.800 (0.544–0.937), 0.722 (0.585–0.798), 0.615 (0.426–0.721), 0.867 (0.703–0.958), and 0.750 (0.574–0.848) in B-mode (28 sites); 0.875 (0.632–0.965), 0.900 (0.706–0.972), 0.875 (0.632–0.965), 0.900 (0.706–0.972), and 0.889 (0.673–0.969) in EUS-EG (18 sites). In the linear instrument, the sensitivity, specificity, PPV, NPV, and accuracy for vascular invasion were 0.600 (0.260–0.873), 0.467 (0.353–0.558), 0.273 (0.118–0.397), 0.778 (0.589–0.929), and 0.500 (0.330–0.636) in B-mode (20 sites); 1.000 (0.610–1.000), 0.900 (0.744–0.900), 0.800 (0.488–0.800), 1.000 (0.826–1.000), and 0.929 (0.705–0.929) in EUS-EG (14 sites).

**Table 2:** Overall results (95% confidence interval)

	Dynamic CT (48 sites)	EUS B-mode (48 sites)	EUS-EG (32 sites)
Sensitivity	0.733 (0.525–0.880)	0.733 (0.524–0.882)	0.917 (0.723–0.983)
Specificity	0.697 (0.602–0.763)	0.606 (0.511–0.673)	0.900 (0.784–0.940)
PPV	0.524 (0.375–0.628)	0.458 (0.327–0.511)	0.846 (0.667–0.908)
NPV	0.852 (0.736–0.933)	0.833 (0.702–0.926)	0.947 (0.825–0.990)
accuracy	0.708 (0.578–0.800)	0.646 (0.515–0.739)	0.906 (0.761–0.956)

*CT* computed tomography, *EUS* endoscopic ultrasonography, *EUS-EG* endoscopic ultrasonography elastography, *PPV* positive predictive value, *NPV* negative predictive value

In the 27 subjects (29 sites) in difficult diagnosis sites group, the sensitivity, specificity, PPV, NPV, and accuracy for vascular invasion were 0.556 (0.303–0.772), 0.750 (0.636–0.847), 0.500 (0.273–0.694), 0.789 (0.670–0.892), and 0.690 (0.533–0.824) on dynamic CT; 0.667 (0.400–0.863), 0.700 (0.580–0.788), 0.500 (0.300–0.647), 0.824 (0.682–0.927), and 0.690 (0.524–0.811) in EUS B-mode; and 0.889 (0.635–0.979), 0.850 (0.736–0.890), 0.727 (0.520–0.801), 0.944 (0.818–0.989), and 0.862 (0.705–0.918) in EUS-EG, respectively. These results show that EUS-EG had the best diagnostic performance (Table 3). For PV (9 sites), SMV (3 sites), and SMA (1 site), the sensitivity, specificity, PPV, NPV, and accuracy for vascular invasion were 0.250 (0.050–0.449), 0.889 (0.800–0.977), 0.500 (0.099–0.898), 0.727 (0.654–0.800), and 0.692 (0.569–0.815) on dynamic CT; 0.500 (0.171–0.817), 0.667 (0.520–0.808), 0.400 (0.136–0.654), 0.750 (0.585–0.909), and 0.615 (0.413–0.811) in EUS B-mode; and 1.000 (0.613–1.000), 0.889 (0.717–0.889), 0.800 (0.490–0.800), 1.000 (0.806–1.000), and 0.923 (0.685–0.923) in EUS-EG, respectively. The diagnoses of all three modalities matched in 12 sites and the diagnostic accuracy was 100% (12/12) in this situation. Meanwhile, the diagnoses of either two out of three modalities (CT and EUS B-mode, CT and EUS-EG, or EUS B-mode and EUS-EG) matched in 5 sites, 7 sites, and 5 sites, and the diagnostic accuracy for each situation was 40% (2/5), 85.7% (6/7), and 80% (4/5), respectively. In difficult diagnosis site group, 9 sites had “an abnormal vessel missing a hyperechoic layer vascular surface” on EUS B-mode. Of these, pathological vascular invasion was positive in 4 sites and negative in 5 sites. EUS-EG successfully diagnosed 8 of the 9 sites (One example was false negative).

**Table 3:** Results in group of difficult diagnosis sites (29 sites) (95% confidence interval)

	dynamic CT	EUS B-mode	EUS-EG
Sensitivity	0.556 (0.303–0.772)	0.667 (0.400–0.863)	0.889 (0.635–0.979)
Specificity	0.750 (0.636–0.847)	0.700 (0.580–0.788)	0.850 (0.736–0.890)
PPV	0.500 (0.273–0.694)	0.500 (0.300–0.647)	0.727 (0.520–0.801)
NPV	0.789 (0.670–0.892)	0.824 (0.682–0.927)	0.944 (0.818–0.989)
Accuracy	0.690 (0.533–0.824)	0.690 (0.524–0.811)	0.862 (0.705–0.918)

*CT* computed tomography, *EUS* endoscopic ultrasonography, *EUS-EG* endoscopic ultrasonography elastography, *PPV* positive predictive value, *NPV* negative predictive value

The 29 sites were divided into arterial (8 sites) and portal groups (21 sites). In the arterial group, the sensitivity, specificity, PPV, NPV, and accuracy for vascular invasion were 1.000 (0.418–1.000), 0.500 (0.306–0.500), 0.400 (0.167–0.400), 1.000 (0.612–1.000), and 0.625 (0.334–0.625) on dynamic CT; 1.000 (0.413–1.000), 0.833 (0.638–0.833), 0.667 (0.275–0.667), 1.000 (0.765–1.000), and 0.875 (0.581–0.875) in EUS B-mode; and 1.000 (0.413–1.000), 0.833 (0.638–0.833), 0.667 (0.275–0.667), 1.000 (0.765–1.000), and 0.875 (0.581–0.875) in EUS-EG, respectively. In the portal group, these results were 0.429 (0185–0.619), 0.857 (0.735–0.952), 0.600 (0.258–0.866), 0.750 (0.643–0.833), and 0.714 (0.552–0.841) on dynamic CT; 0.571 (0.290–0.806), 0.714 (0.573–0.832), 0.500 (0.253–0.705), 0.769 (0.617–0.896), and 0.667 (0.479–0.823) in EUS B-mode; and 0.857 (0.567–0.971), 0.857 (0.712–0.914), 0.750 (0.496–0.850), 0.923 (0.767–0.985), and 0.857 (0.664–0.933) in EUS-EG, respectively (Table 4)

**Table 4:** Results in group of difficult diagnosis sites arterial system (8 sites) and portal vein (21 sites)

		Dynamic CT	EUS B-mode	EUS-EG
Sensitivity	Artery	1.000 (0.418–1.000)	1.000 (0.413–1.000)	1.000 (0.413–1.000)
	Portal vein	0.429 (0.185–0.619)	0.571 (0.290–0.806)	0.857 (0.567–0.971)
Specificity	Artery	0.500 (0.306–0.500)	0.833 (0.638–0.833)	0.833 (0.638–0.833)
	Portal vein	0.857 (0.735–0.952)	0.714 (0.573–0.832)	0.857 (0.712–0.914)
PPV	Artery	0.400 (0.167–0.400)	0.667 (0.275–0.667)	0.667 (0.275–0.667)
	portal vein	0.600 (0.258–0.866)	0.500 (0.253–0.705)	0.750 (0.496–0.850)
NPV	artery	1.000 (0.612–1.000)	1.000 (0.765–1.000)	1.000 (0.765–1.000)
	portal vein	0.750 (0.643–0.833)	0.769 (0.617–0.896)	0.923 (0.767–0.985)
accuracy	artery	0.625 (0.334–0.625)	0.875 (0.581–0.875)	0.875 (0.581–0.875)
	portal vein	0.714 (0.552–0.841)	0.667 (0.479–0.823)	0.857 (0.664–0.933)

*CT* computed tomography, *EUS* endoscopic ultrasonography, *EUS-EG* endoscopic ultrasonography elastography, *PPV* positive predictive value, *NPV* negative predictive value

## Discussion

Vascular invasion in PDAC is a factor in staging that is important for determining the therapeutic strategy and surgical procedure. Elastography has been shown to be useful for qualitative diagnosis of PDAC, but there has been no study of this technique for staging diagnosis. This is the first study that has shown the usefulness of elastography for diagnosis of vascular invasion in patients with PDAC.

Previous studies have shown the sensitivity and specificity is 0.72–0.87 and 0.89–0.93 using EUS, and is 0.58–0.63 and 0.92–0.95 using CT for diagnosis of vascular invasion in PDAC [22–24]. In addition, there is a report showing that the contrast-enhanced EUS using Sonazoid® (Daiichi-Sankyo, Tokyo, Japan) in evaluation of portal vein infiltration has sensitivity of 1.00 and specificity of 0.726–1.00 [25]. Based on these results, the diagnostic results for vascular invasion using EUS are similar or better than those with CT; however, it is sometimes difficult to interpret the EUS findings in the same way among the reviewers because EUS is more subjective compared to CT. In this study, vascular invasion was assessed based on easy-to-read colored band using EUS-EG, which showed a high diagnostic ability with sensitivity of 0.917, specificity of 0.900, and accuracy of 0.906. Regarding the interobserver agreement, EUS-EG showed higher κ-coefficients than EUS B-mode (κ = 0.542 vs. κ = 0.625) with sufficient agreement. These results suggest that evaluating colored band in EUS-EG is an easy and reliable method to diagnose vascular invasion in PDAC.

In the group of difficult diagnosis sites, use of colored band in EUS-EG gave good results for arterial and portal invasion, although there were only a few cases with arterial invasion. The number of subjects in the arterial system is small, it is because a case with suspected invasion of the SMA or celiac artery was assessed as borderline resectable or unresectable.

There are few reports on the difference in diagnostic performance between radial and linear EUS, but it is reported that there is no difference in the accuracy rate [23]. In this study, the results of the radial instrument in B-mode were better than those of the linear type, but the diagnostic ability was improved by adding the EG findings in both types of the EUS scope.

The vascular invasion diagnostic ability of EUS-EG in both artery and portal vein was superior to those of EUS B-mode alone; therefore, the EUS-EG vascular invasion diagnosis using colored band was considered to be objective and useful for assessment of arterial and portal vascular invasion. However, it is difficult to use EUS-EG to assess a tumor that is shown not to be in contact with vessels using CT or EUS B-mode, and lesions in which a tumor and vessel cannot be imaged on the same cross-section by EUS B-mode. Therefore, it is not meaningful to use EUS-EG vascular invasion diagnosis in such cases. The NCCN Guidelines specify that EUS should be performed for selected patients [1]. It is preferable to perform EUS-EG proactively in cases in which diagnosis of vascular invasion using CT is difficult (those with slight contact of the tumor with vessels), such as those defined as difficult diagnosis site in this study. In particular, if the diagnoses of the three modalities coincide, it is highly possible to predict the presence or absence of vascular infiltration.

This study has several limitations. First, it was a retrospective study at a single center. The elastography technique used was not standardized for all patients, and some EUS-EG images mainly performed for tumor characterization were retrospectively reviewed for the evaluation of vascular invasion. Further prospective studies with standardized elastography techniques may be required. Second, endoscopists and devices differed among patients. In our study, we have used 6 different combinations of ultrasound systems and EUS probes, and the use of different machines may have impacted the results. However, it is difficult to evaluate the diagnostic performances for each ultrasound system or probe because of the small number. There have been several reports using different machines in the same study to evaluate the usefulness of EUS-EG [26, 27]. Considering these previous reports and the fact that multiple EUS modalities can be used in the real-world practice, we think it is reasonable to include multiple machines in the present study. Within these limitations, our results may show that EUS-EG is useful for diagnosis of vascular invasion. Further studies and an accumulation of cases are needed to validate EUS-EG for diagnosis of vascular invasion in PDAC.

## Conclusions

Our results for diagnosis of vascular invasion in PDAC suggest that a combination of EUS B-mode with EUS-EG improves diagnostic performance. In particular, cases in which vascular invasion cannot be clearly assessed by dynamic CT should be evaluated using EUS-EG.

## Data Availability

The data of this study are available from the corresponding author upon reasonable request.

## Abbreviations

PDAC: Pancreatic ductal adenocarcinoma
EUS: Endoscopic ultrasound
EUS-EG: Endoscopic ultrasound elastography
CT: Computed tomography
NCCN: National Comprehensive Cancer Network
ROI: Region of interest
PPV: Positive predictive value
NPV: Negative predictive value
PV: Portal vein
SMV: Superior mesenteric vein
SPV: Splenic vein
SMA: Superior mesenteric artery
SPA: Splenic artery
GDA: Gastroduodenal artery
RHA: Right hepatic artery
CI: Confidence interval
